# Donor-Derived Cell-Free DNA Versus Left Ventricular Longitudinal Strain and Strain-Derived Myocardial Work Indices for Identification of Heart Transplant Injury

**DOI:** 10.3390/biomedicines13040841

**Published:** 2025-04-01

**Authors:** Timea Teszak, Timea Barcziova, Csaba Bödör, Lajos Hegyi, Luca Levay, Beata Nagy, Attila Fintha, Adam Szijarto, Attila Kovacs, Bela Merkely, Balazs Sax

**Affiliations:** 1 Heart and Vascular Centre, Semmelweis University, H-1122 Budapest, Hungary; 2 Department of Pathology and Experimental Cancer Research, Semmelweis University, H-1085 Budapest, Hungary; 3 Department of Surgical Research and Techniques, Semmelweis University, H-1122 Budapest, Hungary

**Keywords:** cardiac allograft rejection, donor-derived cell-free DNA, heart transplantation, left ventricular global longitudinal strain, myocardial work

## Abstract

**Background/Objectives:** Donor-derived cell-free DNA (dd-cfDNA) is a marker of graft injury that increases in acute rejection and has excellent negative predictive value. Left ventricular global longitudinal strain (LVGLS) and strain-derived myocardial work indices are novel echocardiographic parameters with growing applications. Still, they have been poorly investigated in heart transplant (HTx) recipients so far. We sought to examine the diagnostic impact of left ventricular longitudinal strain-derived indices in diagnosing myocardial injury as assessed by dd-cfDNA after HTx. **Methods**: Since October 2022, HTx recipients have been shifted from our endomyocardial biopsy (EMB)-based rejection surveillance protocol to a monthly dd-cfDNA-led rejection assessment. We analysed the percentage of donor-derived to total cell-free DNA. For echocardiographic analysis, patient selection was restricted to those transplanted ≥ 6 months. We used 2D speckle-tracking echocardiography to assess LVGLS and strain-derived myocardial work parameters. **Results**: We analysed four hundred and forty-nine dd-cfDNA samples from seventy-one patients until November 2024. The mean dd-cfDNA fraction remained very low (0.13 ± 0.06%). Eighty-eight percent of surveillance EMBs that would have otherwise been performed were avoided. The mean LVGLS was lower than the literature reference values. We found no correlation between dd-cfDNA and LVGLS. Transplanted hearts had different myocardial work indices than the reference values reported in the literature. **Conclusions**: dd-cfDNA effectively rules out clinically significant acute rejection and decreases the need for invasive surveillance EMBs. LVGLS seems less sensitive than dd-cfDNA for the identification of myocardial injury in the early stages of HTx rejection in patients at low risk for rejection.

## 1. Introduction

Endomyocardial biopsy (EMB) is regarded as the standard method for monitoring heart transplant (HTx) rejection [1]. However, it is invasive, associated with procedural complications, and can result in sampling errors. Its histopathologic interpretation shows high interobserver variability [2,3]. With the advancement of immunosuppressive therapy, the yield of routine surveillance EMBs has declined [2,4,5]. Thus, non-invasive tools for HTx rejection surveillance are of great importance.

Previous trials have shown that the donor-derived cell-free DNA (dd-cfDNA) assay has a high negative predictive value to rule out acute HTx rejection. It has been proposed that recipients can be monitored with dd-cfDNA after post-HTx day 28 onwards [1,3,6,7,8,9,10,11].

2D speckle-tracking echocardiography is an emerging echocardiographic modality for evaluating myocardial deformation to detect early subclinical cardiac dysfunction [12,13]. Data show that global and regional left ventricular (LV) longitudinal strain values are lower after HTx than in the control population [14,15]. It is not evident whether the reduced LV longitudinal strain values reflect the “normal” values of the transplanted heart or whether they are signs of subtle alteration in the contractile function of the allograft [15]. The correlation between dd-cfDNA and LV longitudinal strain-derived parameters remains unknown to date. Myocardial work analysis is a novel tool in echocardiography, incorporating LV afterload into global longitudinal strain (GLS) testing. However, the role of myocardial work analysis in the assessment of HTx injury remains mostly unknown to date.

We sought to investigate whether a reduction in longitudinal strain parameters may result from early HTx injury as assessed by dd-cfDNA.

## 2. Materials and Methods

### 2.1. Recruitment of Patients

Consecutive patients at low risk for rejection (*n* = 71) were shifted from our EMB-based rejection surveillance protocol (EMBs performed in year 1 posttransplant) to monthly dd-cfDNA-led assessment and included in this prospective study [8]. Exclusion criteria included multi-organ transplant recipients, a blood transfusion that contained white blood cells within the past 30 days, pregnancy, an EMB performed within the previous 24 h, and EMB-proven rejection episodes, which required intensification of the maintenance immunosuppressive therapy. Furthermore, we identified a cohort of consecutive HTx recipients treated for antibody-mediated rejection (*n* = 9). Informed consent was acquired from each patient, and the study was conducted in accordance with the Declaration of Helsinki.

### 2.2. Collection of Data

We retrospectively collected the clinical data of the patients from the institutional electronic records and saved them in a separate database.

### 2.3. Transthoracic Echocardiography

Experienced operators performed transthoracic echocardiography on the Philips Epiq Elite or Philips Epiq CVx (Philips, Andover, MA, USA) (*n* = 151) or the Vivid E95 (GE HealthCare, Horten, Norway) (*n* = 43) machine with an electrocardiogram continuously traced [16]. Echocardiographic examinations were performed on the same day and after drawing blood for dd-cfDNA measurement. For echocardiographic analysis, patient selection was restricted to those transplanted ≥ 6 months before inclusion to minimise the confounding effect of pretransplant ischemic injury [17]. Left ventricular ejection fraction (LVEF) was calculated using the biplane method of disc summation [16]. For strain analysis, each study utilised the 18-segment model of LV segmentation. Left ventricular global longitudinal strain (LVGLS) and myocardial work indices were calculated offline after automated tracking of myocardial deformation (TomTec Imaging Systems, Munich, Germany). Echocardiographic examinations were excluded if tracking quality was inadequate in >1 segment. Regional longitudinal strain values (LV basal, mid, and apical strain, LV lateral, and septal strain) were assessed. The apical sparing ratio was determined as the apical LV longitudinal strain divided by the sum of the average basal and the average mid LV longitudinal strain. Calculated apical sparing was defined as an apical sparing ratio ≥ 1 [18]. Myocardial work was depicted noninvasively by pressure–strain loop analysis. To assess myocardial work, LV longitudinal strain and non-invasive cuff blood pressure measurement recordings were exported and analysed [19]. The opening and closure timepoints of the mitral and aortic valves were required to set the beginning and the end of the main phases of the cardiac cycle, and they were identified on the apical 3-chamber loop through visual assessment. They were used to dissect the strain curve. The previously published and validated LV reference pressure curve was adjusted according to the cardiac cycle events and the cuff-based peak systolic blood pressure and then was concatenated with the strain curve. The software output displays the indices that depict the pressure–strain loop. Four values were assessed:

1. global work index (GWI): total work performed by the LV during systole,
2. global constructive work (GCW): LV work that contributes to LV ejection and quantifies the energy consumed by the myocardium, which effectively contributes to the cardiac output (shortening during systole and lengthening during isovolumetric relaxation),
3. global wasted work (GWW): LV work that does not lead to LV ejection, and it quantifies the energy that is wasted (lengthening during systole and shortening during isovolumetric relaxation),
4. global work efficiency (GWE): reflects the percentage of myocardial work performed that is translated into cardiac output (i.e., the ratio of constructive and wasted works) [20,21].

### 2.4. Donor-Derived Cell-Free DNA Analysis

We performed the first dd-cfDNA measurement with a simultaneous surveillance EMB in 24 cases (34%). For measurement of dd-cfDNA, 8 mL of whole venous blood was collected into Streck blood collecting tubes (Streck, La Vista, NE, USA). dd-cfDNA analysis was performed in months 2, 3, 4, 5, 6, 7, 8, 10, and 12 after HTx when clinical evaluation, electrocardiogram, echocardiography, and routine blood tests were also obtained. For the patient cohort treated for antibody-mediated rejection, sampling for dd-cfDNA was performed either preceding surveillance EMB or during routine follow-up visits. The percentage of donor-derived to total cell-free DNA was analysed by a commercially available dd-cfDNA test (AlloSeq, CareDx, Inc., Brisbane, CA, USA), which has been proven to accurately quantify dd-cfDNA with high repeatability and reproducibility [22]. The assay was performed by the Illumina MiSeq system (Illumina, Inc., San Diego, CA, USA), as described elsewhere [23]. At each measurement, at least one control sample was assessed with the clinical samples to ensure data consistency across independent measurements. The turnaround time was three working days. A dd-cfDNA level ≥ 0.20% was regarded as injury, while ≥0.35% was considered severe injury [3]. The 0.2–0.25% dd-cfDNA range was considered a grey zone [6].

### 2.5. Endomyocardial Biopsy

We performed a for-cause EMB at each instance when dd-cfDNA results exceeded 0.35%. In the first year of our clinical experience, we performed a for-cause EMB when the first dd-cfDNA result was found to be 0.25–0.35%. If the histopathology did not verify more than mild rejection and the subsequent dd-cfDNA values decreased, we avoided further EMBs [8]. From the second year of our clinical experience onward, in case of no clinical suspicion of rejection, we continued regular dd-cfDNA testing in patients with a dd-cfDNA level of 0.25–0.35%. The International Society for Heart and Lung Transplantation grading scale was used to evaluate EMB specimens [1,24].

### 2.6. Statistical Analysis

Data were expressed as count (percentage), mean ± standard deviation (SD), or median (min–max), as appropriate. The distribution of the dataset was assessed using D’Agostino–Pearson and Shapiro–Wilk tests. Between-group differences were evaluated using the paired *t*-test, Mann–Whitney test, or Wilcoxon matched-pairs signed rank test based on the distribution of the dataset, as appropriate. Different groups were compared by either the ordinary ANOVA test or Kruskal–Wallis test. For correlation analysis, Pearson’s or Spearman’s correlation coefficient was used based on the distribution of the dataset. We considered *p* < 0.05 statistically significant. GraphPad Prism 10.4.0 software (GraphPad Software, Inc., La Jolla, CA, USA) was used for the statistical analyses.

## 3. Results

Seventy-one patients were transitioned from our EMB-based rejection surveillance protocol to dd-cfDNA-led assessment. Four hundred and forty-nine dd-cfDNA samples were analysed in 26 runs from October 2022 until November 2024 (Table 1).

**Figure 1:**
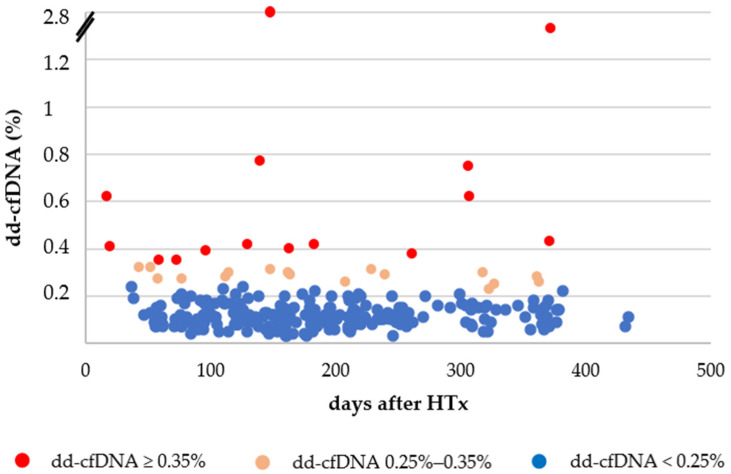
Distribution of donor-derived cell-free DNA (dd-cfDNA) data points in the function of time after heart transplantation (HTx).

The dd-cfDNA fraction remained very low (0.13 ± 0.06%) in stable patients. Nine dd-cfDNA measurements did not pass the quality check. In one case, we performed EMB because the patient was in the early posttransplant period (<4 months after HTx). In the remaining 8 cases, we repeated the dd-cfDNA measurement the following month because overt HTx rejection could be ruled out based on other routine clinical parameters. Owing to elevated dd-cfDNA results suggesting myocardial injury, we performed 26 for-cause EMBs. One EMB proved moderate cellular rejection that required intravenous steroid therapy. Six EMBs showed mild cellular rejection, and one EMB confirmed mild antibody-mediated rejection. Moreover, we performed two for-cause EMBs before the arrival of dd-cfDNA results because of suspicion of rejection based on other clinical parameters [serum N-terminal prohormone of brain natriuretic peptide (NT-proBNP) level, tacrolimus trough level, echocardiography] which confirmed moderate cellular rejection that required intravenous steroid therapy. The patients with EMB-proven rejection were transitioned back to regular EMB testing. Eighteen EMBs showed no rejection: two dd-cfDNA results corresponded to perioperative injury (testing was performed < 1 month posttransplant), while one result was presumably caused by pre-analytical error. Three hundred and ninety-six (88%) routine surveillance EMBs that would have otherwise been performed were avoided (Figure 1).

For echocardiographic analysis, we included 194 echocardiograms of 51 patients. 4 ± 1 echocardiographic examinations were analysed per patient. The median age of the donors was 45 (24–62) years, and 24% were female, whereas the median age of the recipients at the time of imaging was 55 (18–67) years, and 20% were female. Echocardiographic examination was performed at 249 (180–434) days posttransplant. The mean LVEF was preserved (57.2 ± 7.0%). The mean LVGLS, mean apical 4-chamber, 2-chamber, and 3-chamber longitudinal strain values were lower than the reference values reported in the literature (Table 2) [25].

Regarding the regional strain values, we found a significant difference between lateral and septal longitudinal strain values (−17.3 ± 3.6% vs. −12.5 ± 4.1%; *p* < 0.0001). Moreover, we observed a significant difference between basal, mid, and apical longitudinal strain values (−16.2 ± 4.8% vs. −12.7 ± 3.5% vs. −14.9 ± 6.0%; *p* < 0.0001). We found no correlation between LVGLS and dd-cfDNA values (*n* = 156; *p* = 0.762). Additionally, no significant correlation between regional longitudinal strain indices and dd-cfDNA values has been identified. There was no significant difference regarding LVEF, LVGLS, or apical sparing ratio, measured 6 or 12 months after HTx. The regional longitudinal strain values were consistent between the two timepoints except for lateral longitudinal strain, which showed a significant difference between indices measured 6 and 12 months after HTx (−16.6 ± 2.9% vs. −18.6 ± 3.4%; *p* = 0.031) (Table S1).

Six recipients were found to have an apical sparing ratio ≥ 1. We found no difference regarding dd-cfDNA values between patients who presented with apical sparing compared to recipients who did not have an apical sparing ratio ≥ 1.

To analyse myocardial work, we evaluated 59 echocardiograms of 22 HTx recipients. The median time from HTx was 293 (183–402) days. Compared to the results of the EACVI NORRE study in healthy volunteers, the transplanted hearts had lower GWI, GCW, and GWE values and higher GWW values (Table 3) [26]. There was no correlation between any myocardial work parameters and dd-cfDNA values.

We created three subgroups based on dd-cfDNA values: dd-cfDNA < 0.25% (*n* = 171), 0.25–0.35% (*n* = 11), and ≥0.35% (*n* = 7). We found no between-group differences regarding LVEF and LV longitudinal strain indices. However, between-group differences in NT-proBNP values reached statistical significance (Table 4).

Regarding the prespecified ranges of dd-cfDNA < 0.25%, 0.25–0.35%, and ≥0.35%, we found no correlation between dd-cfDNA and LVEF, the strain-derived echocardiographic parameters and serum NT-proBNP level.

In patients with HTx rejection on for-cause EMB (*n* = 10 rejection episodes of 9 patients), the mean dd-cfDNA level rose to 0.77 ± 0.79%. The mean LVEF was preserved (60.8 ± 8.5%). The mean LVGLS was −15.6 ± 5.3%. LVGLS was not pathognomonic in the case of EMB-proven rejection, either.

The prespecified patient cohort treated for antibody-mediated rejection consisted of 9 HTx recipients (4 females; mean age at the time of measurement was 52 ± 10 years). None of them presented with hemodynamic compromise. We analysed 26 dd-cfDNA samples from this patient cohort 40 ± 23 months after HTx. The median dd-cfDNA value was 2.76% (0.68–10.01%). The mean LVEF was preserved (58.8 ± 9.8%). The mean LVGLS was −17.4 ± 3.3%. We found no correlation between dd-cfDNA values and LVEF or LV longitudinal strain parameters.

## 4. Discussion

This study shows, for the first time in the literature, the long-term experience of the clinical use of a standardised and analytically validated local laboratory-run dd-cfDNA assay in routine HTx rejection surveillance. Similarly to previous studies on centralised testing, the dd-cfDNA values remained very low in patients with no overt, clinically significant allograft rejection (Table S2) [3,6,7,11]. The dd-cfDNA-assay effectively rules out clinically significant acute rejection and decreases the need for routine invasive EMBs.

LVEF is generally preserved until severe injury has evolved in the transplanted heart [14,15]. Accordingly, the mean LVEF was preserved in our cohort, and changes in LVEF failed to suggest injury to the transplanted organ, as assessed by dd-cfDNA. However, it is important to consider that there were no episodes of HTx rejection with hemodynamic significance in our patient cohort.

Compared to the LVEF, the LVGLS is more significantly associated with myocardial injury in case of acute cellular rejection (ACR) [27,28]. After HTx, ACR leads to local oedema, inflammatory infiltrate, myocyte damage, and the development of myocardial fibrosis, which can affect the subendocardial myocardial fibre, leading to decreased longitudinal myocardial function assessed by strain analysis [13]. Some studies have shown that the absolute values of LVGLS are lower in HTx recipients with ACR grade ≥ 2R detected in EMB than in patients without significant ACR [13,29]. It has also been proven that repeated episodes of ACR lead to reduced longitudinal systolic function evaluated by left ventricular (LV) longitudinal strain. In HTx recipients with ACR, LVGLS is a significant predictor of mortality [13,29]. There is also data showing that failure to improve longitudinal strain after the third posttransplant month is associated with a higher risk for death, heart failure hospitalisation, and EMB-proven rejection [14]. One study proved that gene expression profiling, a non-invasive tool for rejection surveillance that quantifies immune-related transcriptome expressions, correlated with LV longitudinal strain parameters independently of HTx rejection, cardiac allograft vasculopathy, or other clinical variables [14]. However, other studies showed that LV longitudinal strain values remained attenuated independently of EMB-proven cellular rejection episodes and that global and regional LV longitudinal strain values were lower after HTx than in the control population. It has been proven that HTx recipients possess lower longitudinal strain values than reference values reported in the literature [14,15,30]. We found that the mean LVGLS, mean apical 4-chamber, 2-chamber, and 3-chamber longitudinal strain values were lower than reference values reported in healthy individuals, comparable to previous studies on HTx recipients [14,15,25]. We found that the LVGLS values were consistent and did not change between months 6 and 12 posttransplant (though measured in a small cohort of patients), which underlines the stability of these indices over time. In a previous study, LVGLS values have been described to be very similar from year 1 up to a median of 10.6 years posttransplant [15].

Although apical sparing has been assessed in several clinical scenarios, its role in the serial evaluation of the transplanted heart remains uncertain [31]. In one study, regional longitudinal strain analysis showed a gradient of longitudinal strain values increasing from basal to apical segments. Mean basal and mid segments showed abnormal longitudinal strain parameters, while apical segments were normal [29]. Contrarily, another study found persistently attenuated LV apical longitudinal strain values at year 1 posttransplant [14]. When assessing the regional LV longitudinal strain parameters, we observed consistently decreased mean basal, mid, and apical strain parameters in our HTx patient cohort. However, we found no correlation between the apical sparing ratio and myocardial injury as assessed by dd-cfDNA. We found a statistically significant difference between lateral and septal longitudinal strain values. This finding can be explained by conduction disturbances leading to dyssynchrony, which have long been described in HTx recipients [32].

We found no correlation between any LV longitudinal strain parameters and myocardial injury as assessed by dd-cfDNA. Since dd-cfDNA directly assesses the injury of the transplanted heart, and an elevation in its values could even be observed preceding the diagnosis of EMB-proven HTx rejection, the lack of correlations between either LV longitudinal strain indices and dd-cfDNA could reflect the relative insensitivity of these strain-derived parameters in the diagnosis of early HTx injury [33]. Moreover, several factors decrease the LV systolic function in the posttransplant period. The decrease in LV longitudinal strain parameters could be explained by the surgical procedure itself, pericardiotomy, allograft ischemic time, and donor heart and recipient characteristics [13,32]. Donor variables include advanced age, LV hypertrophy, and gender mismatch. Recipient characteristics comprise alloimmune responses, HTx rejection episodes, and the development of cardiac allograft vasculopathy [14]. In the early posttransplant period, the adaptation and the different positioning of the transplanted heart, early graft dysfunction, pericardial fluid, sepsis, and multi-organ failure may affect these echocardiographic indices [17]. Non-immune risk factors include sympathetic denervation after surgery and conduction abnormalities, which may lead to mechanical dyssynchrony. Other characteristics are hyperlipidemia, hypertension, diabetes mellitus, hyperhomocysteinemia, advanced age, and infections. Myocardial fibrosis is a histopathological finding in HTx recipients, which has been associated with restrictive physiology and advanced cardiac allograft vasculopathy. Neurohormonal activation may accompany HTx rejection episodes, influencing the loading conditions and, thus, the LVGLS [13,14,30,32]. However, as LVGLS has been shown to be stable over time from 6 months posttransplant onwards, it highlights the importance of the continuous complementary measurement of this parameter as part of the non-invasive surveillance of the transplanted heart. The baseline assessment of this value may serve as a reference parameter for future posttransplant complications. The likelihood of allograft rejection increases if several parameters are abnormal in the assessment of HTx recipients [17]. Intraindividual changes may indicate an alteration in the integrity of the allograft and warrant further examinations of the underlying pathology.

The application of LVGLS is limited by its load-dependency. The specific loading conditions include systemic hypertension and neurohormonal activation. Myocardial work analysis incorporates LV afterload into GLS testing; thus, it is less load-dependent than the LVGLS and may provide additional information in the HTx population [30]. Myocardial work has been evaluated in several clinical scenarios to assess the added value of myocardial work compared to LVEF and GLS [20,21,34]. However, data and clinical applications on strain-derived myocardial work indices in the HTx population are sparse. There is emerging data on identifying cardiac allograft vasculopathy, acute rejection, and right ventricular dysfunction [30,35,36]. One study aimed to analyse the association between myocardial work parameters evaluated by echocardiography and acute cellular rejection as assessed by transvenous EMBs among adult HTx recipients in the first posttransplant year. They proved that GWE values increased in recipients diagnosed with ≥ISHLT grade 2R cellular rejection [30]. One study explored the right ventricular functional adaptation after HTx and derivated the right ventricular myocardial work index. Pulmonary artery pressure was invasively measured during right heart catheterisation. HTx rejection was marked by a significantly reduced myocardial work index, which was an independent predictor of EMB-proven rejection-related myocardial damage [36]. One study aimed to assess the correlations between LV myocardial work indices and the presence of CAV in a paediatric HTx population. Paediatric HTx patients underwent coronary angiography within three months of their echocardiogram. Echocardiograms were post-processed with speckle-tracking echocardiography, and myocardial work indices were derivated. This study showed that decreased GWE values correlated with paediatric cardiac allograft vasculopathy [35].

It has been proven that myocardial work parameters after HTx significantly differ from the results of the EACVI NORRE study in healthy volunteers [32]. Similarly to previous studies, we found that transplanted hearts had lower GWI, GCW, and GWE values and higher GWW values than the results of the EACVI NORRE study in healthy volunteers [30,32]. The reduced LVGLS values of the transplanted hearts could explain this, as described earlier (i.e., surgical factors, ischaemic time, myocardial fibrosis, conduction abnormalities, donor heart and recipient characteristics, and mismatch) [13,14,17,32]. Conduction abnormalities may lead to mechanical dyssynchrony and, thus, explain the decreased GWE values and the increased GWW values [32]. We found no correlation between myocardial work parameters and dd-cfDNA values (though measured in a small cohort of patients). Badano et al. pointed out that the likelihood of allograft rejection increases if several parameters are abnormal in the assessment of HTx recipients. When an abnormality is detected, a careful revision of the present and baseline study images is recommended [17]. The intraindividual changes in myocardial work parameters may indicate an alteration in the integrity of the allograft and may warrant further examinations of the underlying pathology. The clinical utility and the role of strain-derived myocardial work indices in diagnosing HTx injury require further investigation.

Our study has some limitations. The single-centre nature of the study, the low number of echocardiographic examinations (we excluded a considerable number of examinations as baseline echocardiographic assessment was recommended to be assessed six months after HTx), and subgroup analyses, such as the evaluation of myocardial work indices, are based on smaller cohorts that limited further analyses and the generalizability of these findings [17].

## 5. Conclusions

This study demonstrates for the first time the long-term experience of a standardised and analytically validated local laboratory-run dd-cfDNA assay for HTx rejection surveillance in the routine clinical practice. It effectively rules out clinically significant acute rejection and decreases the need for routine invasive EMBs. The LVGLS seems less sensitive than the dd-cfDNA assay for the identification of myocardial injury in the early stages of graft injury in HTx recipients at low risk for rejection. The clinical utility of strain-derived myocardial work indices in identifying HTx injury requires further investigation.

## Figures and Tables

**Table 1:** Patient characteristics.

Variable	Population
Number of patients	71
Number of samples	449
Pre-transplant diagnosis	
Non-ischemic cardiomyopathy	39
Ischemic cardiomyopathy	24
Hypertrophic cardiomyopathy	5
Arrhythmogenic right ventricular dysplasia	1
Valvular heart disease	1
Restrictive cardiomyopathy	1
Female sex (%)	18 (25%)
Mechanical support at the time of heart transplantation	
Durable circulatory support (%)	4 (6%)
Temporary circulatory support (%)	3 (4%)
Age at time of heart transplantation [median (min–max); years]	54 (18–67)
Days posttransplant at enrolment [median (min–max); days]	62 (17–367)
Enrolment without simultaneous endomyocardial biopsy (%)	47 (66%)
Immunosuppression	
Tacrolimus/mycophenolate/methylprednisolone (%)	68 (96%)
Cyclosporine/mycophenolate/methylprednisolone (%)	1 (1%)
Tacrolimus/mycophenolate (%)	2 (3%)
Tacrolimus trough level at enrolment [median (min–max); (ng/mL)]	13.5 (3.0–23.7)
Patients presenting with donor-specific antibody at enrolment (%)	14 (20%)
Maximum MFI	1492 (737–15,771)
NT-proBNP level at enrolment [median (min–max); (pg/mL)]	1271 (193–35,000)
Creatinine level at enrolment [median (min–max); µmol/L]	106 (57–709)

Abbreviations: MFI, mean fluorescent intensity; NT-proBNP, N-terminal prohormone of brain natriuretic peptide.

**Table 2:** Two-dimensional echocardiographic parameters of left ventricular longitudinal strain (*n* = 194 echocardiograms of 51 patients) compared to the results of the EACVI NORRE study (*n* = 549, one echocardiogram per patient).

	Study Examinations Mean ± SD	EACVI NORRE Mean ± SD
longitudinal strain, %		
apical 4-chamber	−16.0 ± 3.3	−22.6 ± 3.0
apical 2-chamber	−14.8 ± 4.2	−23.2 ± 3.3
apical 3-chamber	−15.4 ± 3.5	−21.6 ± 3.2
LVGLS	−15.6 ± 2.8	−22.5 ± 2.7

Abbreviations: LVGLS, left ventricular global longitudinal strain; SD, standard deviation.

**Table 3:** Strain-derived myocardial work parameters (*n* = 59 echocardiograms of 22 patients) compared to the results of the EACVI NORRE study (*n* = 226, one echocardiogram per patient).

	Study Examinations Mean ± SD	EACVI NORRE Mean ± SD
GWI (mmHg%)	1420.3 ± 361.8	1896 ± 308
GCW (mmHg%)	1790.5 ± 425.6	2232 ± 331
GWW (mmHg%)	142 (16–954)	78.5 (53–122.2)
GWE (%)	93 (70–99)	96 (94–97)

Abbreviations: GWI, global work index; GCW, global constructive work; GWW, global wasted work; GWE, global work efficiency; SD, standard deviation.

**Table 4:** Two-dimensional echocardiographic parameters of left ventricular longitudinal strain, left ventricular ejection fraction, and serum N-terminal prohormone of brain natriuretic peptide levels in the function of prespecified ranges of donor-derived cell-free DNA (*n* = 194 echocardiograms of 51 patients).

	dd-cfDNA < 0.25% Mean ± SD	dd-cfDNA 0.25–0.35% Mean ± SD	dd-cfDNA ≥ 0.35% Mean ± SD	*p*
Longitudinal strain, %				
apical 4-chamber	−16.0 ± 3.2	−16.0 ± 3.4	−17.6 ± 4.8	NS
apical 2-chamber	−14.8 ± 4.3	−14.5 ± 4.2	−14.8 ± 3.6	NS
apical 3-chamber	−15.4 ± 3.5	−15.4 ± 4.3	−17.3 ± 3.2	NS
LVGLS	−15.6 ± 2.7	−15.3 ± 3.5	−16.6 ± 3.7	NS
lateral strain	−17.2 ± 3.5	−16.6 ± 3.5	−19.4 ± 4.0	NS
septal strain	−12.6 ± 3.5	−12.9 ± 3.7	−15.0 ± 4.2	NS
basal strain	−16.2 ± 4.8	−16.3 ± 5.3	−16.3 ± 4.6	NS
mid strain	−12.6 ± 3.5	−13.1 ± 4.1	−13.3 ± 3.6	NS
apical strain	−14.7 ± 5.9	−14.1 ± 5.7	−19.4 ± 8.1	NS
LVEF, %	57.1 ± 7.0	56.1 ± 5.2	63.2 ± 6.2	NS
NT-proBNP, pg/mL	1451 ± 4212	952 ± 971	1855 ± 1667	0.044

Abbreviations: dd-cfDNA, donor-derived cell-free DNA; LVEF, left ventricular ejection fraction; LVGLS, left ventricular global longitudinal strain; NS, not significant; NT-proBNP, N-terminal prohormone of brain natriuretic peptide; SD, standard deviation.

## Data Availability

The original contributions presented in this study are included in the article and Supplementary Materials. Further inquiries can be directed to the corresponding author.

## Supplementary Materials

The following supporting information can be downloaded at: https://www.mdpi.com/article/10.3390/biomedicines13040841/s1, Table S1: Left ventricular longitudinal strain values and left ventricular ejection fraction measured 6 and 12 months after heart transplantation. Table S2: Donor-derived cell-free DNA values in patients with no heart transplant rejection.
