# Supplementary material for: Donor-Derived Cell-Free DNA Versus Left Ventricular Longitudinal Strain and Strain-Derived Myocardial Work Indices for Identification of Heart Transplant Injury
Source: Biomedicines. 2025 Apr 1;13(4):841. doi: 10.3390/biomedicines13040841 (PMC12025175; doi:10.3390/biomedicines13040841)
Supplement: Supplementary file 1 [file biomedicines-13-00841-s001.zip › biomedicines-3401063-supplementary.pdf]

Supplementary Material

Table S1. Left ventricular longitudinal strain values and left ventricular ejection fraction measured 6 and 12 months after heart transplantation.

	**6 months after HTx mean ± SD n = 20**	**12 months after HTx mean ± SD n = 20**	***p***
longitudinal strain, %			
apical 4-chamber	−16.0 ± 3.1	−17.6 ± 3.7	NS
apical 2-chamber	−14.7 ± 3.6	−15.7 ± 3.6	NS
apical 3-chamber	−14.8 ± 3.4	−16.1 ± 3.4	NS
LVGLS	−15.2 ± 2.4	−16.5 ± 2.9	NS
lateral strain	−16.6 ± 2.9	−18.6 ± 3.4	0.031
septal strain	−11.7 ± 3.0	−11.9 ± 7.1	NS
basal strain	−15.4 ± 4.4	−16.0 ± 3.9	NS
mid strain	−12.4 ± 2.9	−13.9 ± 3.4	NS
apical strain	−14.3 ± 5.6	−16.8 ± 4.8	NS
apical sparing ratio	0.53 ± 0.21	0.58 ± 0.21	NS
**LVEF, %**	56.9 ± 7.1	58.7 ± 8.3	NS

Abbreviations: HTx, heart transplantation; LVEF, left ventricular ejection fraction; LVGLS, left ventricular global longitudinal strain; NS, not significant; SD, standard deviation.

Table S2. Donor-derived cell-free DNA values in patients without heart transplant rejection.

Study	Assay	median dd-cfDNA (%)
DOAR [1]	AlloSure (targeted NGS)	0.07
GRAfT [2]	Shotgun sequencing	0.03
DEDUCE [3]	Prospera (targeted NGS)	0.04
FreeDNA-CAR [4]	Allonext (targeted NGS)	0.095
Heart and Vascular Centre, Semmelweis University	AlloSeq (targeted NGS)	0.11

Abbreviations: dd-cfDNA, donor-derived cell-free DNA.

References

1. Khush, K.K.; Patel, J.; Pinney, S.; Kao, A.; Alharethi, R.; DePasquale, E.; Ewald, G.; Berman, P.; Kanwar, M.; Hiller, D.; et al. Noninvasive detection of graft injury after heart transplant using donor-derived cell-free DNA: A prospective multicenter study. *Am J Transplant* **2019**, *19*, 2889-2899, doi:10.1111/ajt.15339.
2. Agbor-Enoh, S.; Shah, P.; Tunc, I.; Hsu, S.; Russell, S.; Feller, E.; Shah, K.; Rodrigo, M.E.; Najjar, S.S.; Kong, H.; et al. Cell-Free DNA to Detect Heart Allograft Acute Rejection. *Circulation* **2021**, *143*, 1184-1197, doi:10.1161/CIRCULATIONAHA.120.049098.
3. Kim, P.J.; Olymbios, M.; Siu, A.; Wever Pinzon, O.; Adler, E.; Liang, N.; Swenerton, R.; Sternberg, J.; Kaur, N.; Ahmed, E.; et al. A novel donor-derived cell-free DNA assay for the detection of acute rejection in heart transplantation. *J Heart Lung Transplant* **2022**, *41*, 919-927, doi:10.1016/j.healun.2022.04.002.
4. Jimenez-Blanco, M.; Crespo-Leiro, M.G.; Garcia-Cosio Carmena, M.D.; Gomez Bueno, M.; Lopez-Vilella, R.; Ortiz-Bautista, C.; Farrero-Torres, M.; Zegri-Reiriz, I.; Diaz-Molina, B.; Garcia-Romero, E.; et al. Donor-derived cell-free DNA as a new biomarker for cardiac allograft rejection: A prospective study (FreeDNA-CAR). *J Heart Lung Transplant* **2024**, doi:10.1016/j.healun.2024.11.009.
